# Challenges Facing Antimicrobial Stewardship Programs in the Endemic Region for Coccidioidomycosis

**DOI:** 10.1093/ofid/ofae041

**Published:** 2024-01-24

**Authors:** Justin F Hayes, David E Nix

**Affiliations:** Division of Infectious Diseases, University of Arizona, Tucson, Arizona, USA; Valley Fever Center for Excellence, University of Arizona, Tucson, Arizona, USA; Valley Fever Center for Excellence, University of Arizona, Tucson, Arizona, USA; Department of Pharmacy Practice and Science, University of Arizona, Tucson, Arizona, USA

**Keywords:** antifungal agents, antifungal stewardship, antimicrobial stewardship, coccidioidomycosis, diagnostic stewardship

## Abstract

Coccidioidomycosis poses a significant cost and morbidity burden in the United States. Additionally, coccidioidomycosis requires constant decision-making related to prevention, diagnosis, and management. Delays in diagnosis lead to significant consequences, including unnecessary diagnostic workup and antibacterial therapy. Antifungal stewardship considerations regarding empiric, prophylactic, and targeted management of coccidioidomycosis are also complex. In this review, the problems facing antimicrobial stewardship programs (ASPs) in the endemic region for coccidioidomycosis, consequences due to delayed or missed diagnoses of coccidioidomycosis on antibacterial prescribing, and excess antifungal prescribing for prevention and treatment of coccidioidomycosis are elucidated. Finally, our recommendations and research priorities for ASPs in the endemic region for coccidioidomycosis are outlined.

## Teaser

Coccidioidomycosis requires constant decision-making related to prevention, diagnosis, and management. Diagnostic challenges result in excess antibacterial and antifungal prescriptions, significantly impacting antimicrobial stewardship efforts. Greater awareness and specific guidance are needed.

Antimicrobial resistance is a global threat. A comprehensive analysis incorporating data from >200 countries and territories concluded that bacterial resistance contributed to an estimated 4.95 million deaths in 2019, with 1.27 million deaths estimated to be directly attributable to bacterial resistance [1]. Implementation of antimicrobial stewardship programs (ASPs) has received increased focus and has been endorsed as a strategy to mitigate the problem of resistance [2–4]. A significant driver of unnecessary antibiotic therapy includes conditions that mimic bacterial infections [5–7]. One such mimicker is coccidioidomycosis.

Coccidioidomycosis is a fungal infection caused by *Coccidioides immitis* or *Coccidioides posadasii* that is endemic in the southwestern United States, Mexico, Central America, and South America [8]. A pulmonary syndrome is the primary presentation with coccidioidomycosis being a common cause of community-acquired pneumonia (CAP) in endemic locations [9–11]. This resembles the presentation of bacterial CAP, and awareness to order testing for coccidioidomycosis should occur for patients presenting with CAP who have resided in or traveled to the endemic region. Diagnosis of coccidioidomycosis typically occurs through serologic testing with enzyme immunoassay (EIA), immunodiffusion (IDF), and complement fixation (CF), the currently available testing methodologies. EIAs normally possess higher sensitivity, faster turnaround times, and lower costs compared to IDF and CF, whereas IDF and CF possess higher specificity compared to EIAs, with availability normally limited to reference laboratories or larger academic medical centers [12, 13]. Criteria for diagnosis of coccidioidomycosis are outlined by the European Organization for Research and Treatment of Cancer and Mycoses Study Group Education and Research Consortium, with proven disease requiring histopathology or direct microscopy of specimens obtained from an affected site demonstrating characteristic forms (ie, spherules) or growth of *Coccidioides* spp and probable disease requiring exposure to endemic areas, clinical signs compatible with disease, and positive antibody testing in either the serum or cerebrospinal fluid [14].

Ultimately, issues affecting timely diagnosis of coccidioidomycosis, treatment of coccidioidomycosis, and prevention aspects related to coccidioidomycosis have a significant impact on ASPs in the endemic region and should be understood by the broader infectious diseases (ID) and mycology community. In this review, these complex issues are described in detail. Finally, our recommendations and research priorities for ASPs in the endemic region for coccidioidomycosis are outlined.

## SCOPE OF THE PROBLEM

Disease resulting from coccidioidomycosis is quite diverse and may mimic infection due to other bacterial, viral, and fungal pathogens. Failure to accurately label the disease leads to unnecessary diagnostic procedures and inappropriate antibacterial and antifungal therapy. The diagnostic challenges for ASPs in the endemic region for coccidioidomycosis are in large part shaped by 3 interconnected issues: (1) failure to consider diagnosis of coccidioidomycosis in uncomplicated pulmonary coccidioidomycosis, (2) delays in diagnosis of complications or disseminated coccidioidomycosis disease, and (3) lack of appreciation for the consequences of missed or delayed diagnosis.

In Tucson and Phoenix, Arizona, approximately a quarter of CAP cases are due to coccidioidomycosis [9, 10]. Unfortunately, despite coccidioidomycosis being a common cause of CAP in endemic areas, assessments of CAP testing practices have consistently shown a lack of awareness of and testing for coccidioidomycosis [15–19]. In one study, Chang and colleagues assessed CAP testing practices from 2 health systems in the Phoenix metropolitan area. The results demonstrated that coccidioidomycosis testing took place in only 2% and 13% of patients presenting with CAP at the 2 health systems, respectively [15]. In another study, an evaluation of patients presenting with CAP to emergency departments in Arizona demonstrated that only 2.8% of CAP patients were tested for coccidioidomycosis [16]. Finally, in a Southern California–based analysis, Tartof et al utilized a cohort study to analyze patterns around coccidioidomycosis testing for patients presenting with CAP. The cohort consisted of 33 756 individuals and demonstrated that only 6% were tested for coccidioidomycosis within 1 year of CAP diagnosis. Interestingly, among individuals who initially tested negative for coccidioidomycosis, only 5% were retested within 30 days and 8% were retested within 90 days [17].

In addition to lack of recognition of uncomplicated pulmonary coccidioidomycosis, complications and disseminated coccidioidomycosis are underrecognized. In one report, a 40-patient case evaluation revealed an average diagnostic delay of 12 weeks (range, 8–16 weeks) for cutaneous coccidioidomycosis, with many cases initially diagnosed as bacterial cellulitis [20]. Experts describe the clinical spectrum and frequency of coccidioidomycosis as approximately 60% asymptomatic, 30% uncomplicated primary, 5% fibrocavitary complications, and <1% extrapulmonary dissemination [21]. While immunocompromised hosts with deficits in cell-mediated immunity have risk for severe coccidioidomycosis, specific host variants are also implicated as drivers of dissemination [22]. This challenges clinicians to understand that more severe presentations of coccidioidomycosis occur in both immunocompetent and immunocompromised hosts and to remain judicious when it comes to the diagnostic workup and management. Among severely immunocompromised patients, the underlying condition may limit procedures to obtain tissue samples for diagnosis, and use of serologic testing has reduced sensitivity related to immunosuppression [23, 24].

In addition to lack of awareness for both nonsevere and severe presentations of coccidioidomycosis, analyses of healthcare utilization in the endemic region have revealed the consequences due to delays in diagnosis. Specifically, the analyses have revealed increased cost burden due to unnecessary healthcare services including antimicrobial therapy, laboratory tests, imaging, procedures, and hospitalizations [25–27]. In a large analysis, an Arizona-based health system analyzed coccidioidomycosis practice patterns over 3 years. The study demonstrated that for 2043 coccidioidomycosis diagnoses, 72.9% of the diagnoses were made during a hospital admission with 40.6% of these patients requiring neither intensive care unit support or a hospital-requiring procedure. The effects on antimicrobial prescribing were substantial, revealing that for 95.4% of the diagnosed patients in the hospital, 13 135 orders were written in 2017, 16 755 orders written in 2018, and 23 355 orders written in 2019. Notably, anti–methicillin-resistant *Staphylococcus aureus* agents (ie, vancomycin, linezolid, and daptomycin) as well as fluoroquinolones were prescribed in large numbers [27]. Table 1 provides a summary of studies assessing antimicrobial prescribing in coccidioidomycosis-endemic areas. The aggregate data overwhelmingly demonstrate a significant amount of unnecessary antibacterial prescribing. From an antimicrobial stewardship perspective, earlier diagnosis of coccidioidomycosis could mitigate misdirected diagnostic procedures and inappropriate management including antibacterial prescribing.

**Table 1.** Studies Demonstrating Effects on Antimicrobial Prescribing in Coccidioidomycosis-Endemic Areas

Publication	Study Design	Effects on Antimicrobial Prescribing	Take-Home Points
Donovan et al [25]	Retrospective study of patients at Banner University Medical Center–Tucson (1 Jan 2015–18 Sep 2017) with selected *ICD-9/10* codes. This information was used to determine initial symptom presentation date and official CM diagnosis date to quantify the delay in diagnosis. Antibacterial prescriptions were characterized as antibacterial medication orders submitted prior to CM diagnosis date.	Antibacterial prescriptions ordered prior to CM diagnosis (N = 276 patients): 1103 antibacterial prescriptions ordered prior to diagnosis. Vancomycin and daptomycin responsible for 22% of the orders.	Single-center retrospective analysis demonstrating that 43% of 276 patients with CM had a delay in diagnosis >1 mo Median delays in diagnosis ranged between 17 and 54 d 1103 antibacterial prescriptions orders submitted prior to diagnosis of CM
Pu et al [27]	Retrospective study analyzing CM practice patterns in a large Arizona-based health system over 3 y (2017–2019). This included data from 15 hospitals, 53 primary care sites, and 48 urgent care sites. Patients identified with CM *ICD-10* code and ordered CM antibodies.	Quantification of antibacterial prescriptions: For 95.4% of 1491 hospital-diagnosed patients: 13 135 antibacterial prescriptions ordered in 2017, 16 755 antibacterial prescriptions ordered in 2018, and 23 355 antibacterial prescriptions ordered in 2019. Median number of antibacterial prescription orders per patient: Not requiring ICU care or a procedure during admission: 14 orders per patient; ICU care but no procedure during admission: 21 orders per patient; procedure but no ICU care during admission: 21 orders per patient; ICU care and procedure during admission: 41 orders per patient. Overall—19 orders per patient.	Large amount of CM diagnoses made only after hospital admission Large amount of antibacterial prescribing occurred during these hospitalizations Study demonstrates a practice pattern of not considering the diagnosis of CM in ambulatory settings
Tartof et al [17]	Cohort study of all patients in a large health system who were both diagnosed with CAP and received outpatient treatment. The study evaluated for CAP diagnoses from all care settings and documentation of a CM *ICD-9* code from 1 wk prior to 1 wk after sample collection date for confirmed CM cases.	Having antibacterial agents prescribed ≥2 times from 1 wk prior to CAP visit to first CM test: Associated with increased odds of positive CM testing (aOR, 4.57 [95% CI, 1.29–16.12]). Total number of times prescribed additional antibacterials before/on follow-up CAP visits to 1 y after CAP diagnosis with or without CM testing (N = 33 756 patients): 0 prescriptions: 29 248 patients 1 prescription: 3607 patients 2 prescriptions: 669 patients 3 prescriptions: 161 patients ≥4 prescriptions: 71 patients	Study highlights opportunities to reduce antibacterial prescribing in endemic regions for CM by increasing awareness to test for CM in patients presenting with CAP Patients with confirmed cases of CM were more likely to receive multiple courses of antibacterials
Chi et al [28]	Retrospective study that investigated CM testing and treatment patterns through use of EHR in a large integrated health network.	Antibacterial patterns (N = 530 patients): 70% of patients received antibacterials 3 mo prior to first positive CM test; 36% of patients received antibacterials 3 mo after first positive CM test. Median antibacterial prescriptions: Patients who received antibacterials had a median of 3 prescriptions (IQR, 2–7). Antifungal patterns (N = 530 patients): 14% received antifungals the year prior to first positive CM test; 79% patients received antifungals after first positive CM test. Median time from first positive CM test to antifungal prescription: 7 d (IQR, 2–13). Event sequence: Most common event sequence was (1) antibacterial prescription, (2) positive CM testing, (3) antifungal prescription.	In this study, most patients received antibacterials prior to first positive CM test After positive CM testing, most patients received antifungal prescriptions, while antibacterial prescribing decreased Awareness for testing in patients with CAP could reduce antibacterial prescribing

Abbreviations: aOR, adjusted odds ratio; CAP, community-acquired pneumonia; CI, confidence interval; CM, coccidioidomycosis; EHR, electronic health record; *ICD-9*, *International Classification of Diseases, Ninth Revision*; *ICD-10*, *International Classification of Diseases, Tenth Revision*; ICU, intensive care unit; IQR, interquartile range.

Ultimately, surveillance for coccidioidomycosis is a critical issue that has an overarching impact on both the lack of diagnosis of coccidioidomycosis and full appreciation of the consequences of delayed diagnosis. In 2019, the Centers for Disease Control and Prevention (CDC) reported 20 061 coccidioidomycosis cases from 23 of 27 states and jurisdictions where coccidioidomycosis is reportable, with 97% cases reported from Arizona and California [29]. The CDC acknowledged that the data do not provide additional elements, such as exposure history, treatments provided, type of presentation, and severity of illness. In addition, cases are underreported. Only 50 000 of the estimated 150 000 coccidioidomycosis cases annually present for medical attention [8]. Initial testing for coccidioidomycosis involves serologic testing (ie, EIA, IDF, CF), which is insensitive early in the disease and insensitive due to anergy in immunocompromised patients [30]. McCotter and colleagues argue for enhanced surveillance including better capture of populations at risk, increased use of genomic epidemiologic methods, and expansion of reporting to all states [31].

## EFFECTS ON ANTIFUNGAL STEWARDSHIP PRACTICE

### Antifungal Prescribing Overview

In addition to the challenges ASPs face regarding antibacterial prescribing, antifungal stewardship is heavily influenced by being in a region endemic for coccidioidomycosis. Two studies have shed light on outpatient antifungal prescribing trends [32, 33]. First, Benedict and colleagues examined the IQVIA Xponent database (which captures 92% of all retail prescriptions in the United States) for antifungal prescriptions in 2018. The results demonstrated that fluconazole was the most frequently prescribed antifungal (75% of all antifungal prescriptions), with overall antifungal prescribing showing regional variability and Arizona demonstrating higher fluconazole prescribing rates compared to some of the other Western states [32]. Second, Al-Obaidi et al investigated antifungal prescribing patterns from a Medicare Part D provider database during 2013–2020. The results again showed regional variability. Interestingly, the Southern region demonstrated the highest total days’ supply of antifungal prescriptions regionally, but at the state level, Arizona had the highest total days’ supply for all antifungal azoles except voriconazole in 2020 [33]. In terms of inpatient fluconazole prescribing, our own experience has shown that 63.5% of the usage is dedicated to either empiric, targeted, or prophylaxis management of coccidioidomycosis [34]. Additional work evaluating empiric coccidioidomycosis prescribing demonstrated that of 106 patients, 25 (23.6%) ultimately had a positive coccidioidomycosis test during the inpatient workup despite prior receipt of empiric coccidioidomycosis treatment (J. F. H., unpublished data).

Coccidioidomycosis requires constant decision-making related to prevention, diagnosis, and management. Although there is debate about whether antifungal treatment initiation is necessary for uncomplicated coccidioidomycosis pneumonia [8, 35, 36], the current Infectious Diseases Society of America guidelines for coccidioidomycosis suggest that antifungal treatment should be considered for illness requiring hospitalization. Fluconazole at a dose of 400 mg daily or itraconazole at a dose of 200 mg twice daily given for 3–6 months is advised for management of coccidioidomycosis by current guidelines [8]. Due to its lower cost, predictable absorption, and fewer drug interactions when compared to other azoles, fluconazole is favored for coccidioidomycosis management [37].

### Coccidioidomycosis Prophylaxis

Prophylaxis in immunocompromised populations is an additional consideration for fluconazole use in endemic areas. This mainly occurs in the solid organ transplant (SOT) population due to concern for the risk of either reactivation or de novo infection with coccidioidomycosis [38]. In patients with a history of symptomatic coccidioidomycosis or positive serology prior to transplant, lifelong prophylaxis is a strong consideration. Organ donors’ coccidioidomycosis history is also considered, particularly for lung transplant candidates. In other scenarios, there are individuals who develop positive serology posttransplant without clear evidence of disease. With these individuals undergoing immunosuppression, development of serious coccidioidomycosis infection due to reactivation or possibly new exposure carries a significant mortality risk. This has been investigated in multiple studies [39–43]. One transplant center in the endemic region noted that while a targeted prophylaxis protocol decreased infection rates in SOT recipients, infections still resulted. Higher rates of dissemination and mortality in this patient population were particularly concerning [37]. The cumulative data led to current guidance, which is to provide prophylaxis to patients undergoing organ transplantation who reside or have resided in the endemic area for 6–12 months posttransplant. If a patient has a history of coccidioidomycosis or positive serology at the time of transplant, lifelong secondary prophylaxis is recommended [8, 44]. Fluconazole prophylaxis for coccidioidomycosis in other at-risk and immunosuppressed populations, such as individuals on biologic response modifiers, is not as well described or established.

### Fluconazole Toxicity

With the extensive use of fluconazole in endemic locations as well as the duration of therapy required for management of coccidioidomycosis, fluconazole toxicity is an important consideration. Although fluconazole adverse events are usually not life-threatening, hepatotoxicity and cardiac toxicity can occur. In addition, other side effects, such as alopecia, xerosis, and cheilitis have been frequently described [37, 45, 46]. In one study, Davis and colleagues described fluconazole long-term effects through retrospective evaluation of 124 adult patients with proven or probable coccidioidomycosis treated with fluconazole for a prolonged period. More than half (51.6%) of the patients experienced an adverse event, with 65.6% of the patients experiencing an adverse event requiring therapeutic modification [47]. Further work examining the consequences of prolonged antifungal therapy, particularly high-dose fluconazole, in patients on therapy or prophylaxis for coccidioidomycosis is needed. Fluconazole poses a risk precipitating drug interactions due to inhibition of CYP3A4, CYP2C9, and CYP2C19 enzymes. Many of the interactions were evaluated with low doses of fluconazole (50–100 mg), and the significance of using higher doses (400–800 mg/day) is not well defined [48, 49]. There is risk of ventricular arrythmias in patients with inherited long QT syndrome or use in combination with other drugs that prolong the QTc [50, 51].

### Nonfluconazole Therapy

Despite fluconazole being favored for coccidioidomycosis management, it consistently possesses higher minimum inhibitory concentrations (MICs) than voriconazole, itraconazole, and posaconazole based on in vitro susceptibility testing [52–55]. In one analysis, a susceptibility assessment of 581 *Coccidioides* spp isolates sent to a referral fungal testing laboratory was performed. The results demonstrated that 37.3% of the isolates demonstrated a fluconazole MIC ≥16 μg/mL, while elevated MICs for other triazoles and amphotericin B were not common [55]. However, the significance of in vitro susceptibility results remains unclear given the differences in growth patterns and morphology under different growth conditions. There is no evidence that clinical efficacy is greater for newer azoles despite lower MICs. Given the prevalence of coccidioidomycosis, patients not responding to fluconazole or experiencing fluconazole intolerance are commonly encountered, leading to use of newer azoles such as voriconazole, posaconazole, and isavuconazonium sulfate for salvage therapy. Comparative data from clinical trials are lacking [37, 56–58]. Amphotericin B formulations are mainly reserved for severe and/or refractory cases [59].

### Antifungal Shortages

An important aspect of ASPs is mitigating drug shortages. Anti-infective drug shortages have become notable in recent years including during the coronavirus disease 2019 (COVID-19) pandemic [60]. At our center, we faced a challenging scenario during the pandemic due to a global shortage of lipid-associated amphotericin B [61]. Amphotericin B is typically reserved for the most severe and refractory coccidioidomycosis cases; however, the shortage required careful evaluation and discussion of each prescription for liposomal amphotericin B. At the same time, there were suspected and proven cases of other fungal infections, such as mucormycosis, for which amphotericin B is critical and lifesaving. To get through the shortage, our ASP discussed the situation and formulated consensus recommendations for our hospital regarding in which instances amphotericin B should be reserved (Table 2). Leaders of the ASP (J. F. H., D. E. N.) brought these recommendations to our ID physician group during a previously scheduled meeting due to the critical nature of the shortage. More recently, we faced a shortage of certain dosage formulations of fluconazole. As noted previously, fluconazole is favored therapy for coccidioidomycosis management, and a shortage brings unique concerns to clinicians practicing in endemic areas, such as increased expense as well as elevated risk of adverse effects (eg, mineralocorticoid excess, phototoxicity, periostitis) due to use of alternative agents [33, 62, 63].

**Table 2.** Our Hospital's Antimicrobial Stewardship Program Consensus Recommendations During Amphotericin B Shortage

Therapy for suspected or documented invasive mucormycosis Therapy for invasive fungal infection in patients with no alternative options Therapy for coccidioidomycosis during the first trimester of pregnancy Initial therapy for severe coccidioidomycosis Limit empiric usage to 72 h in high-risk^a^ patients (case-by-case basis)

^a^High-risk indicates patients diagnosed with hematologic malignancy or recipients of hematopoietic stem cell transplant with worsening on anti-mold prophylaxis, and solid organ transplant recipients with recent treatment for rejection and worsening on anti-mold prophylaxis.

### Incorporation of Coccidioidomycosis Into Antifungal Stewardship Guidance

From a broader antifungal stewardship perspective, a group of international experts published core guidance on antifungal stewardship that established a framework for the development of an antifungal stewardship program either within an existing ASP or as a separate entity [64]. In addition, a white paper dedicated to antimicrobial stewardship in SOT recipients was published with guidance regarding antifungal stewardship [65]. Both publications mainly focused on invasive candidiasis and aspergillosis, but there is a great need for incorporation of additional measures and surveillance in regions endemic for fungal pathogens like *Coccidioides* spp. In addition, diagnostic pathways have significant applicability to optimization of antifungal stewardship [64, 66, 67], and the literature previously discussed in this review strongly suggests that additional focus on diagnostic stewardship would be beneficial.

## OUR RECOMMENDATIONS FOR ANTIMICROBIAL STEWARDSHIP PROGRAMS IN THE ENDEMIC REGION FOR COCCIDIOIDOMYCOSIS

Current guidance on best practice for ASPs mainly comes from the CDC's core elements for hospital antimicrobial stewardship programs [2]. In addition, the CDC has disseminated core elements for outpatient antimicrobial stewardship [68]. In line with these goals, our ASP has focused on preauthorization, prospective audit and feedback, and facility-specific treatment guidelines with particular emphasis on facility-specific guidance. Our program is co-led by an ID physician (J. F. H.) and ID pharmacist (D. E. N.) with current staffing for the program including allocation of 0.8 physician full-time equivalents (FTE) and 1.75 ID pharmacy FTE to support approximately 800 beds.

**Figure 1.**
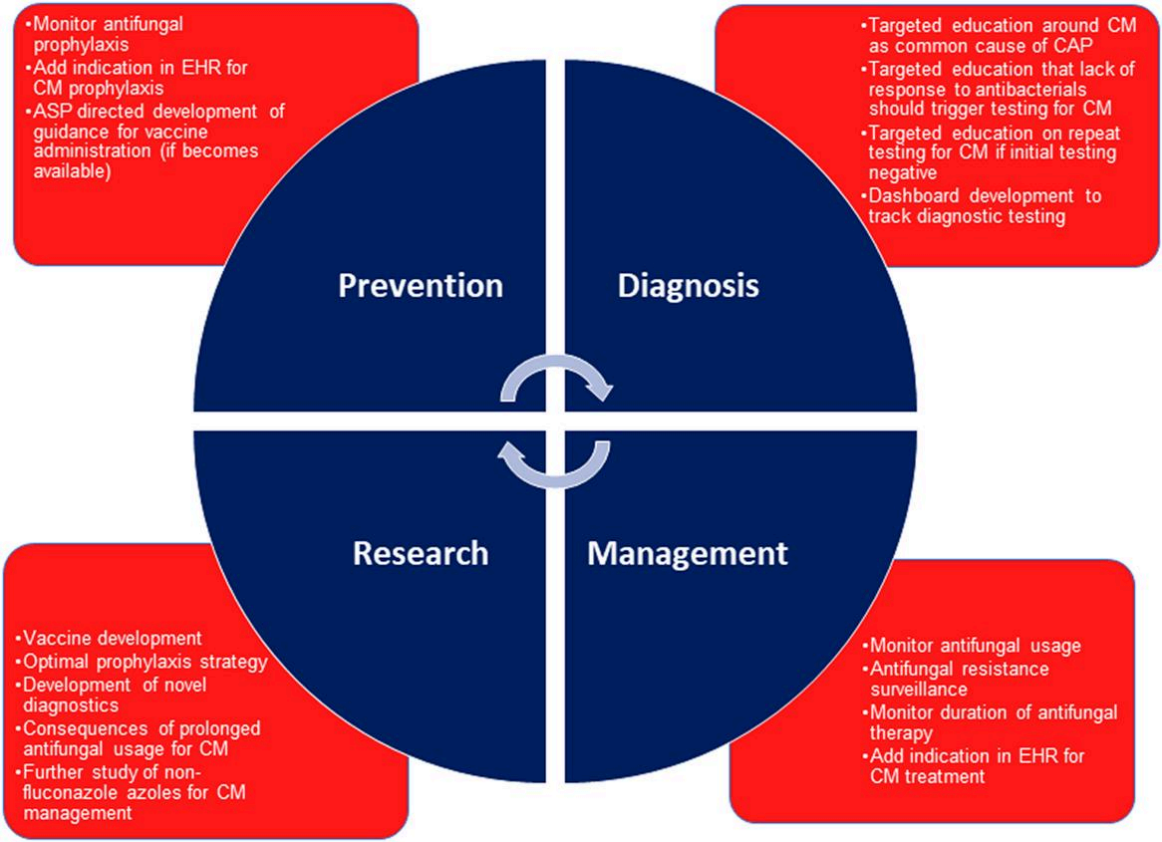
Recommendations for antimicrobial stewardship programs in the endemic region for coccidioidomycosis. The figure illustrates recommendations for stewardship focused on prevention, diagnosis, and management of coccidioidomycosis. Research priorities are also included. Abbreviations: ASP, antimicrobial stewardship program; CAP, community-acquired pneumonia; CM, coccidioidomycosis; EHR, electronic health record.

With location in an endemic region for coccidioidomycosis, our ASP efforts have additional focus. Specifically, our stewardship efforts incorporate focus around the prevention, diagnosis, and management of coccidioidomycosis to identify additional interventions. Figure 1 contains our recommendations and research priorities for ASPs located in the endemic region for coccidioidomycosis, with additional descriptions below. Finally, this section will conclude with a specific example from the urgent care setting.

### Prevention

Focus of an ASP located in the endemic region should incorporate prevention aspects by targeting antifungal prophylaxis. Specifically, monitoring fluconazole usage is important, and identifying usage related specifically to coccidioidomycosis prophylaxis is helpful. This can be done through the electronic health record (EHR) by adding coccidioidomycosis prophylaxis as an indication for azole ordering. There are already initiatives from regulatory agencies, such as The Joint Commission, advocating for use of appropriate indications for antimicrobial prescriptions [69]. Additionally, as mentioned earlier, guidance has been established for the SOT population and coccidioidomycosis prophylaxis. From a research standpoint, previous work has focused on measuring cellular immunity for coccidioidomycosis by using whole blood or isolated lymphocytes, similar to the interferon-γ release assay for latent infection with *Mycobacterium tuberculosis* [70, 71]. In the future, efforts to study the ability of such testing to elucidate the most at-risk individuals undergoing transplantation would be useful to develop a more targeted prophylaxis strategy as opposed to universal antifungal prophylaxis. Additionally, a vaccine is currently under development with the hope that the vaccine will enter human trials and provide another option for prevention [72].

### Diagnosis

Advocacy for integration of diagnostic stewardship within ASPs is currently being conducted [73], and diagnostic stewardship is a perfect fit for ASPs located in the endemic region for coccidioidomycosis. Dashboards are one tool utilized to enhance ASP efficiency [74], and trending coccidioidomycosis diagnostic testing with CAP diagnoses is a strategic area due to coccidioidomycosis as a common cause of CAP in endemic locations. Targeted education is crucial. Our recommendations include instructions that patients presenting with CAP should undergo serologic testing for coccidioidomycosis, lack of response to antibacterials should trigger coccidioidomycosis testing, and a lower threshold for repeat testing (if initial testing is negative) is warranted for immunocompromised hosts and individuals with persistent symptoms and lack of diagnosis. In terms of research, there is need for improved test sensitivity, especially when testing is done early after symptom onset. Moreover, serologic testing is often negative in immunosuppressed patients including SOT recipients and persons receiving immunosuppressant medications [23, 24]. Further development of nucleic acid amplification tests and antigen testing is encouraged.

### Management

Management of coccidioidomycosis overlaps with both prevention and diagnosis of coccidioidomycosis. Fluconazole is favored therapy for management, but other triazoles are sometimes prescribed. Monitoring of antifungal usage regularly and, at a minimum, quarterly is critical to monitor practice trends. Due to the increased needs for antifungal usage, surveillance for azole resistance of other fungal pathogens (ie, *Candida* spp, *Aspergillus* spp) is important and recommended as well. As mentioned earlier, amphotericin B is less commonly used for coccidioidomycosis management, but due to toxicities, careful monitoring is warranted. At our center, the ASP restricts amphotericin B to ID physicians. From an empiric management perspective, patients are sometimes placed on empiric therapy for coccidioidomycosis while awaiting testing results. Additionally, some patients may be discharged from the hospital on fluconazole or another azole with results of testing pending. The ability to characterize the level of empiric coccidioidomycosis prescribing and duration of therapy given to monitor trends is important practice for ASPs in the endemic region. Adding coccidioidomycosis as an indication for treatment, in addition to prophylaxis, is recommended to track trends of antifungal prescribing. In terms of future research, the burden of prolonged antifungal therapy, especially high-dose fluconazole, for coccidioidomycosis is an area worth further exploration.

### Urgent Care Example

The urgent care setting has been a target for ASP interventions and quality improvement research [75–77]. In a prime example, Stenehjem et al implemented a quality improvement initiative across a large urgent care network at Intermountain Healthcare focused on antibiotic prescribing for respiratory conditions [76]. The initiative included a multipronged approach with targeted education, EHR tools, a transparent clinician benchmarking dashboard, and various media (eg, television, newspaper, commitment posters). The study included a 12-month baseline, intervention, and sustainability period. The results demonstrated a 22% reduction in antibiotic prescribing during the first month of implementation with resultant monthly 5% antibiotic prescription decreases during the rest of the intervention period. Data from the sustainability period demonstrated that the improvement remained stable [76]. In similar fashion, a large urgent care network in our health system has been the setting for collaboration with the University of Arizona Valley Fever Center for Excellence (VFCE) for a quality improvement initiative that has potential ramifications for antimicrobial stewardship. The initiative was focused on improving early recognition of coccidioidomycosis in the ambulatory setting with the hope of preventing worsening illness, hospital admissions, and multiple courses of antibacterial therapy due to delayed diagnosis. The intervention consisted of targeted education for new urgent care hires and experienced providers as well as periodic reminder emails and presentations about the importance of early recognition of coccidioidomycosis. A 3-fold increase in coccidioidomycosis testing as a proportion of both visits and patients occurred after implementation, which was significant. Additionally, performing repeat testing if initial testing was negative increased during the study, which is a key target of educational efforts by members of the VFCE [11]. Further work is being performed to sustain and enhance the increased rates of testing with a new dashboard modeled around CAP diagnoses [78]. Future work could enable ASP personnel to measure outpatient antimicrobial prescriptions, including azole antifungals in the urgent care network, to evaluate if the intervention has been successful from a stewardship perspective while defining further target areas for improvement.

## CONCLUSIONS

ASPs have garnered increased support and attention due to the scourge of antimicrobial resistance. In the endemic region for coccidioidomycosis, ASPs are challenged to innovate and develop specific recommendations around the complexities involved in the prevention, diagnosis, and management of coccidioidomycosis. Optimization of antifungal prophylaxis, earlier diagnosis to limit unnecessary antimicrobial prescribing, and increased understanding of the burden of prolonged antifungal usage are critical targets for ASPs in the endemic region. Future directions and research priorities include development of a vaccine, novel diagnostics, and further understanding of the consequences of long-term fluconazole therapy.

## Notes

***Patient consent.*** This review does not contain factors necessitating patient consent.
